# A review of the earthworm *Amynthasmasatakae* (Beddard, 1892) (Clitellata, Megascolecidae), with designation of two new synonyms

**DOI:** 10.3897/BDJ.12.e119599

**Published:** 2024-05-09

**Authors:** Chih-Han Chang, Huei-Ping Shen, Emma Sherlock, Csaba Csuzdi

**Affiliations:** 1 Department of Life Science and Institute of Ecology and Evolutionary Biology, National Taiwan University, Taipei, Taiwan Department of Life Science and Institute of Ecology and Evolutionary Biology, National Taiwan University Taipei Taiwan; 2 Taiwan Biodiversity Research Institute, Ministry of Agriculture, Nantou, Taiwan Taiwan Biodiversity Research Institute, Ministry of Agriculture Nantou Taiwan; 3 Natural History Museum, London, United Kingdom Natural History Museum London United Kingdom; 4 Department of Zoology, Eszterházy Károly Catholic University, Eger, Hungary Department of Zoology, Eszterházy Károly Catholic University Eger Hungary

**Keywords:** earthworm, *Amynthasmasatakae*, *Amynthasrobustus*, *Amynthastriastriatus*, taxonomy

## Abstract

Correct and timely identification of an invasive species during quarantine or at an early stage of invasion before establishment or spread is critical for preventing biological invasions. However, taxonomic confusion of potential invasive earthworm species caused by incorrect taxonomic treatment or reckless taxonomic work has made it difficult to properly recognize potential invasion threats. Through analyzing publicly available DNA sequences of the mitochondrial cytochrome *c* oxidase subunit I (COI) gene, we confirmed the validity of the specific status of *Amynthasmasatakae* (Beddard, 1892), a peregrine earthworm species in East Asia with the potential to spread to other regions of the world, and designated two new synonyms of *A.masatakae*: *Amynthastralfamadore* Blakemore, 2012 syn. nov. and *Amynthasscaberulus* Sun and Jiang, 2021 syn. nov. Additionally, the name *A.triastriatususualis* Dong, Jiang, Yuan, Zhao and Qiu, 2020 is nomenclaturally unavailable since it was published in an electronic journal without ZooBank registration and an explicit statement establishing a new nominal taxon. Specimens described under this unavailable name actually belong to *A.masatakae*. Inadequate literature review and erroneous species identities associated with sequences in GenBank have caused even more problems in the already confusing earthworm taxonomy.

## Introduction

The invasion of non-native earthworms is one of the main threats to forest ecosystems around the world (Bohlen et al. 2004, Frelich et al. 2019, Chang et al. 2021). These belowground invaders have tremendous impacts on the litter layer and soil, altering habitats of microbes, plants and other invertebrates and changing carbon biogeochemistry and nutrient dynamics (Frelich et al. 2019, Chang et al. 2021). In the last two decades, two taxonomic groups have become a main concern among ecologists, conservation biologists and land managers: lumbricid earthworms of European and pheretimoid earthworms of East and Southeast Asian origins.

While there have been ample studies focusing on European invasive earthworms, from their taxonomic identity to ecological impacts, studies focusing on invasive pheretimoid earthworms, especially their taxonomy, are scarce (e.g. Chang et al. (2016)), making species identification and confirmation of potential invasion challenging. For instance, among the 16 invasive pheretimoid species in North America (Chang et al. 2016), one species, *Amynthascarnosus* (Goto and Hatai, 1899), was confirmed only recently (Carrera-Martínez and Snyder 2016, Chang et al. 2016), and the confirmation was made possible only after Blakemore’s revisionary studies several years earlier (Blakemore 2012b, Blakemore 2013b). In contrast, due to the lack of taxonomic information of *A.carnosus* in literature, an earlier record of the species in Taiwan reported in 2005 is now known to be incorrect (Shen et al. 2005). The case of *A.carnosus* documented above highlights the importance of taxonomic studies as the foundation for our understanding on earthworm invasion around the world.

Similar to *A.carnosus* in its distribution and invasion status, *Amynthasmasatakae* (Beddard, 1892) is a cosmopolitan species found in Japan, Korea, China and Taiwan, and has the potential to spread to other regions of the world. However, our current knowledge on *A.masatakae*, both morphological and genetic, does not allow correct identification of this species by most researchers, making confirmation of potential invasion a challenging task. *Amynthasmasatakae* had long been regarded as a junior synonym of *Amynthasrobustus* (Perrier, 1872) (Ljungström 1972, Easton 1981, Blakemore 2003, Tsai et al. 2009, Blakemore 2010, Blakemore 2012a) until Blakemore (2012c) restored the specific status of *A.masatakae* and designated its lectotype and paralectotype. Meanwhile, Blakemore (2012c) described a new species, *Amynthastralfamadore*, based on a specimen collected in South Korea after comparing its morphology and/or DNA barcode with *A.robustus*, *A.masatakae* and *Amynthastriastriatus* (Chen, 1946). The last species, *A.triastriatus*, is an earthworm endemic to China. Its original description is based on a single specimen found in Mt. Omei, Sichuan, central China (Chen 1946). *Amynthastriastriatus* is morphologically similar to *A.masatakae* and *A.robustus*, but can be distinguished from the other two species by having lower numbers of setae. Recently, Dong et al. (2020) discovered two genetic lineages, A and B, within *A.triastriatus*, and based on a 6.3% divergence in the mitochondrial cytochrome *c* oxidase subunit I gene and certain morphological differences, they named the lineage B *Amynthastriastriatususualis*, while considering lineage A as *Amynthastriastriatustriastriatus*. Recently, a new species, *Amynthasscaberulus* Sun and Jiang, 2021, morphologically similar to *A.tralfamadore*, was described from Sichuan and Hunan, central China (Sun et al. 2021). However, no comparison was made between the two taxa (Sun et al. 2021). Additionally, neither Dong et al. (2020) nor Sun et al. (2021) cited relevant studies by Blakemore (2012c), Blakemore (2013a) and Blakemore and Lee (2013), adding new pieces into the already-perplexing puzzle of the identity of *A.masatakae*.

Historically, *A.masatakae* was only reported once in Taiwan (Chuang and Chen 2002). In 2012, we collected seven specimens of *A.masatakae*in eastern Taiwan. These new specimens provide us with a unique opportunity to re-visit the status of this species in recent literature and the associated DNA barcodes reported in those studies. Our goal is to provide a solid and easy-to-use foundation for future identification of this cosmopolitan species.

## Material and methods

### Sample collection and preservation

Earthworms were collected throughout Taiwan during 2005–2019. The specimens were anesthetized in 10% ethyl alcohol and then preserved in 95% ethyl alcohol. They are deposited in the earthworm collection at the Taiwan Biodiversity Research Institute, Jiji, Nantou, Taiwan. The following specimens were selected for phylogenetic analysis (Table 1): One of the seven specimens of *A.masatakae* collected near Chilai mountain house at an elevation of 1380 m in Hualien, eastern Taiwan (24°02'24.93''N, 121°20'51.36''E) on 7 Aug. 2012 (voucher number: East567); eight specimens of *A.robustus* collected during 2005–2019 from six locations (voucher numbers: laut1–laut3, East348, East382, East422, East686 and PT001); five specimens of *Amynthasgracilis* (Kinberg, 1867) collected during 2007–2012 from five locations (voucher numbers: Gra1, Gra2, MTS5, East617 and LLS43) and one specimen of *Perionyxexcavatus* Perrier, 1872 collected from Hualien, eastern Taiwan on 28 Jul. 2011 (voucher number: East341). Specimen of East567 was dissected dorsally and examined under a Leica MZ6 stereo microscope.

In addition to the newly-collected specimens, we examined the morphology of the type specimens of *A.masatakae* archived in the Natural History Museum in London, UK (BMNH 1904.10.5 912-3) on 14 May 2011 (by CHC) and 23 Jul. 2014 (by HPS).

### DNA extraction, polymerase chain reaction and DNA sequencing

Muscle tissues were taken from the posterior 10–20 segments of the specimens and then preserved in 95% ethyl alcohol at -20^o^C. DNA extraction was conducted using the Tissue Genomic DNA Extraction Mini Kit (Favorgen Biotec, Pingtung, Taiwan). Polymerase chain reaction for COI was carried out using the primers LCO1490 and HCO2198 (Folmer et al. 1994) in a 25-μl total volume with 1 cycle at 94 °C for 1 min, followed by 6 cycles of denaturation for 30 s at 94 °C, annealing for 30 s at 45 °C, and extension for 50 s at 72 °C, and then by 30 cycles of denaturation for 30 s at 94 °C, annealing for 30 s at 54 °C, and extension for 50 s at 72 °C, with a final cycle at 72 °C for 10 min. Sequencing was performed using the ABI PRISM BigDye Terminator Cycle Sequencing Ready Reaction Kit, V3.1 and analyzed on an ABI 3730 XL DNA analyzer (Applied Biosystems, CA, USA).

### DNA barcode analysis

COI sequences of *A.masatakae*, *A.robustus*, *A.scaberulus*, *A.triastriatus*, *Amynthasaspergillum* (Perrier, 1872), *Amynthascorticis* (Kinberg, 1867), *A.gracilis*, *Metaphirecalifornica* (Kinberg, 1867) and *Metaphireschmardae* (Horst, 1883) were retrieved from GenBank including those of *A.masatakae* from Kyushu, Japan and *A.robustus* from the Ryukyus, Japan recently reported by Sato et al. (2023). Those of *A.masatakae* and *A.tralfamadore* published by Blakemore (2012c), Blakemore (2013a) and Blakemore and Lee (2013) were not available in GenBank, but could be found as part of the text in the paper. They were also included in the analysis (Table 2). For *A.triastriatus*, Dong et al. (2020) reported 17 COI haplotypes out of 65 sequences. Since sequences of the same haplotype are the same, we selected one or two sequences from each of the 17 COI haplotypes whenever they are available in GenBank. Our search found that a total of 24 sequences in Dong et al. (2020) were missing and five haplotypes (haplotypes 2, 8, 12, 14 and 17) were each represented by one of the missing sequences only, leaving us 13 sequences of 12 haplotypes for subsequent analysis (Table 3). *Perionyxexcavatus* was used as the outgroup.

Sequences were aligned using the default settings of ClustalX 2.0 (Larkin et al. 2007). The alignment was straightforward since the 658-bp region designated as the DNA barcode has no indels. A Maximum Likelihood analysis was conducted using IQ-TREE (Nguyen et al. 2015), with partitions and the most appropriate models of nucleotide substitutions selected with ModelFinder (Kalyaanamoorthy et al. 2017). The robustness of clades was evaluated using ultrafast bootstrap (Hoang et al. 2018) with 1000 pseudo-replicates. Genetic distances were calculated using the uncorrected *p*-distance as implemented in MEGA11 (Tamura et al. 2021).

### Abbreviations used in text and figures

BMNH = Natural History Museum, London, UK

CN = China

IND = India

JP = Japan

KR = Korea

TW = Taiwan

## Data resources

The sequences obtained in this study are available under GenBank accession numbers OR801241–OR801255 (Table 1).

## Results

### Taxonomic review of A.masatakae, A.robustus, A.tralfamadore, A.scaberulus and A.triastriatus

As mentioned earlier, *A.masatakae* was regarded as a junior synonym of *A.robustus* for a long time (Ljungström 1972, Easton 1981, Blakemore 2003, Tsai et al. 2009, Blakemore 2010, Blakemore 2012a). The type locality of *A.masatakae* is Japan (Beddard 1892), while types of *A.robustus* are from Mauritius and Manila of the Philippines (Perrier 1872). Both *A.masatakae* and *A.robustus* share similar characters on body size, setal and segment numbers, number and position of spermathecal pores and structures of diverticulum, caecum, seminal vesicles and accessory glands (Table 4). In Taiwan, the occurrence of *A.masatakae* was first reported by Chuang and Chen (2002) in which this species was found at an elevation of around 1000 m at Mt. Dong-Yan, an agricultural site for temperate fruits since the 1980s in northern Taiwan.

**Figure 1.**
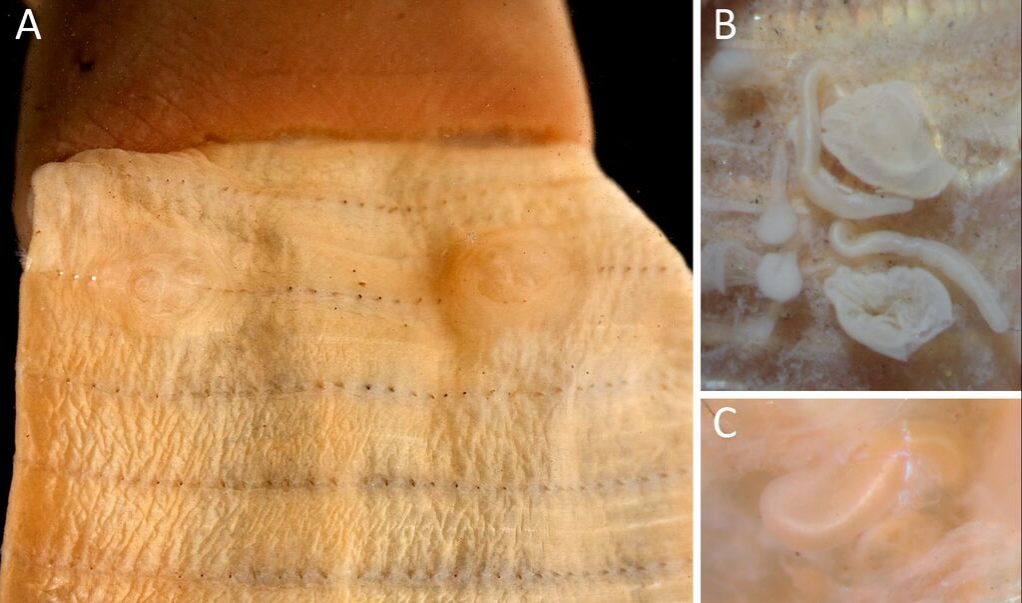
Photos of *Amynthasmasatakae* (Beddard, 1892), syntypes (BMNH 1904.10.5 912-3). **A** Ventral view of clitellum and male pore regions; **B** Spermathecae; **C** Prostatic duct.

Blakemore (2012c) restored the specific status of *A.masatakae* and designated its lectotype and paralectotype according to specimens described by Beddard (1892) and deposited at the Natural History Museum, London, UK (BMNH 1904.10.5 912-3) (Fig. 1). The external and internal characters of *A.masatakae* are as those illustrated by Blakemore (2012c). Meanwhile, Blakemore (2012c) described *Amynthastralfamadore*, based on a specimen collected from an indoor greenhouse designed to reproduce Gotjawal, a forest unique to Jeju Island, at the National Institute of Biological Resources’ Gotjawal Conservatory Exhibition in Incheon, South Korea. Blakemore (Blakemore 2012c: p. 144) states that distinctive characteristics of *A.tralfamadore* are the shape of spermathecae with the diverticula bulb spherical rather than elongate as in *A.robustus* or “paprika-shaped” in *A.masatakae* and its inner face of intestinal caeca perhaps more rugose than in *A.masatakae* types. Moreover, its paired sets of markings in 18 are slightly wider apart on each side compared to those in the *A.masatakae* types (Blakemore 2012c: p. 144). Blakemore (Blakemore 2012c: p. 144) compared its COI sequence with those available from GenBank and found it is only 94% identical to “*Amynthasrobustus*” from Japan (AB542533) and “*Amynthastriastriatus*” from China (EF077538) (Note: "EF077538" is a typo and should be corrected to EF077537 as seen in Fig. 1 in Blakemore and Lee (2013)) and 85% identical to “*A.robustus*” from Taiwan (DQ224191). Later, Blakemore (Blakemore 2013a: p. 30 and p. 35) documented one and three specimens of *A.masatakae* and *A.tralfamadore*, respectively, collected from Cheonji-yeon Falls Park, Jeju, South Korea on 13 Jun. 2012 and suggested the provenance of both species from Jeju. Blakemore (2013a) and Blakemore and Lee (2013) provided DNA barcodes of *A.masatakae* from fresh material collected in Japan and Korea and showed that the sequences were 100% identical to “*A.triastriatus*” from China (EF077537). However, Blakemore (Blakemore 2013a: p. 31) claimed that it is not certain whether the identification as *A.triastriatus* (Chen, 1946: 97) proper is correct and, similarly, *A.robustus* (Perrier, 1872) proper is not proven from Japan (nor Taiwan) and concluded that these GenBank vouchers should likely be recorded as *A.masatakae*.

*Amynthastriastriatus* (Chen, 1946) is an earthworm endemic to China. Its original description is based on a single specimen found in Mt. Omei, Sichuan, central China (Chen 1946). *Amynthastriastriatus* resembles *A.masatakae* in having two pairs of spermathecal pores in 7/8/9, two papillae medial to each male pore, long diverticulum, four pairs of hearts in X–XIII and prostates with a stout duct only (Chen 1946). However, *A.triastriatus* is discernible from *A.masatakae* (and *A.robustus* as well) in its lower setal number, which is not more than 40 even in the post-clitellar region (Table 4). Recently, Dong et al. (2020) reported two lineages, A and B, of *A.triastriatus*. According to a 6.3% COI gene divergence and certain morphological differences between the two lineages, Dong et al. (2020) named lineage B *Amynthastriastriatususualis* without reasoning why lineage A was regarded as *Amynthastriastriatustriastriatus*. On the other hand, *A.triastriatususualis* is dissimilar to the original description of *A.triastriatus* by Chen (1946) concerning the lower setal number in the post-clitellar region (Table 4). Sun et al. (2021) described *Amynthasscaberulus* collected from Sichuan and Hunan, central China. *Amynthasscaberulus* is fairly similar to *A.tralfamadore* in body size, setal and segment numbers, number and position of spermathecal pores, number and arrangement of papillae medial to each spermathecal and male pore and structures of diverticulum, prostate and accessory gland (Table 4). However, no comparison was made between the two taxa (Sun et al. 2021) and neither Dong et al. (2020) nor Sun et al. (2021) cited studies by Blakemore (Blakemore 2012c, Blakemore 2013a) and Blakemore and Lee (2013).

### DNA barcode analysis

**Figure 2.**
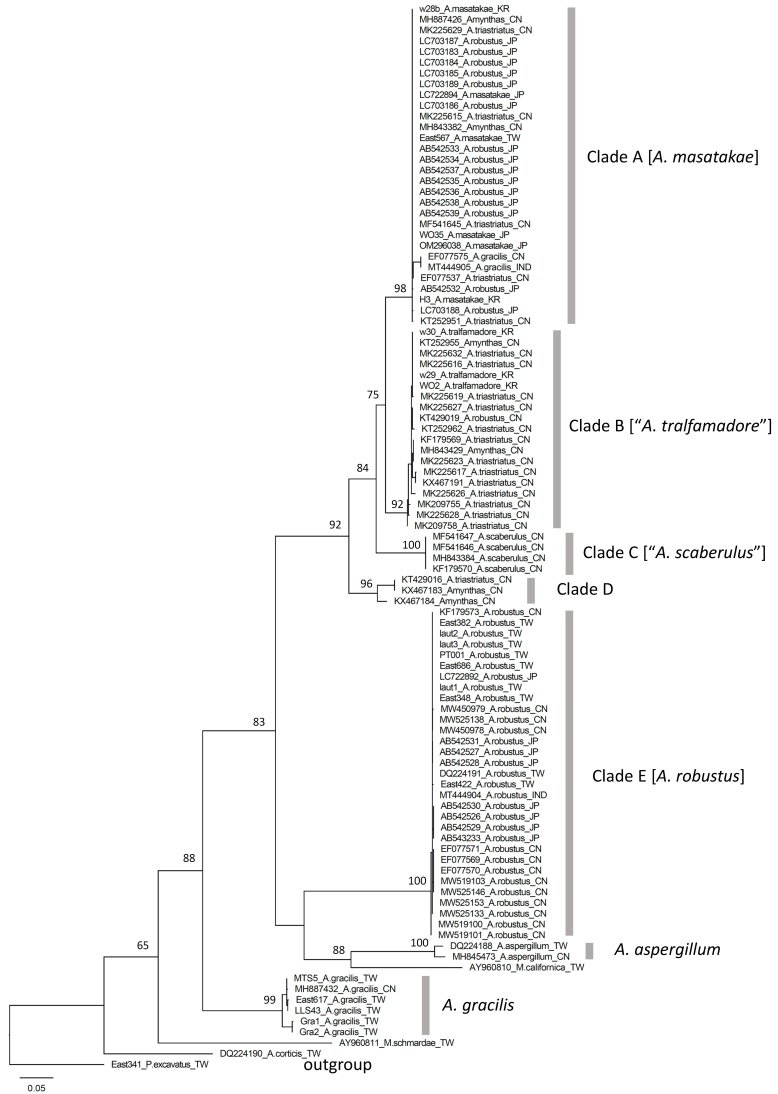
Phylogenetic tree inferred from the Maximum Likelihood analysis of the DNA sequences of the cytochrome *c* oxidase subunit I (COI) gene. Sequences acquired in this study and those available from Blakemore (2012c), Blakemore (2013a) and Blakemore and Lee (2013) were labelled with their voucher numbers. Sequences retrieved from GenBank were labelled with their GenBank accession numbers. All voucher and accession numbers were followed by their scientific names and then by their localities. Numbers around nodes are ultrafast bootstrap values. Scientific names in quotation marks are names synonymized with *Amynthasmasatakae* in this study.

Five apparent clades among our target species/sequences, clades A–E, can be superficially recognized in the COI tree (Fig. 2). Clades A–D form a monophyletic group containing specimens identified as *A.masatakae* by us and by Blakemore (Blakemore 2013a, Blakemore and Lee 2013), as *A.tralfamadore* by Blakemore (Blakemore 2012c, Blakemore 2013a), as *A.triastriatus* by Dong et al. (2020) and as *A.scaberulus* by Sun et al. (2021). Clade E comprises specimens from Taiwan identified by us as *A.robustus*, as well as specimens from India, Japan and China, identified as *A.robustus* by various researchers.

Clade A includes *A.masatakae* specimens from Korea (w28b and H3) and Japan (WO35) reported by Blakemore (2013a) and Blakemore and Lee (2013) and voucher specimen East 567 collected by us from Taiwan. Thus, this clade undoubtedly contains the true *A.masatakae*. Clade B contains all the specimens recorded as *A.tralfamadore* from Korea (WO2, w29 and w30) published by Blakemore (2012c) and Blakemore (2013a). Additionally, clades A and B correspond to lineages B and A of *A.triastriatus*, respectively, in Dong et al. (2020) and contain all specimens reported as *A.triastriatus* in that study. Clades A and B form a highly supported monophyletic group, with a mean *p*-distance of 6.2% between the two clades (Table 5).

Clade C consists of sequences from type specimens of *A.scaberulus* from China (Fig. 2). The *p*-distance between clades C and A and clades C and B are 9.4% and 9.2%, respectively. Clade D is composed of one sequence identified as *A.triastriatus* and two sequences with unknown identity from China. Clades A–D form a monophyletic group, with a *p*-distances of 9.7%, 10.0%, and 11.3% between clade D and clades A, B and C, respectively (Table 5).

In addition to the sequences/specimens mentioned above, 36 sequences in clades A and B clearly came from misidentification, including 16 sequences, one from China and 15 from Japan, identified as *A.robustus*, 18 sequences from China identified as *A.triastriatus* and two sequences, one from China and the other from India, identified as *A.gracilis* (Fig. 2).

## Discussion

### Revision of the taxonomy of A.masatakae

When applying integrative taxonomic criteria widely used in pheretimoid earthworms (Chang and Chen 2005, Chang et al. 2007, Chang and James 2011, Shen et al. 2016, Shen et al. 2022), the low COI-based genetic distance between clades A and B and the monophyly of the two groups strongly indicate that the two clades belong to the same species, i.e., *A.masatakae*. This conclusion is consistent with that of Dong et al. (2020), who treated their lineages B and A, corresponding to clades A and B in our study, as members of the same species. However, Dong et al. (2020) incorrectly considered clade B as *A.triastriatus* and gave clade A a new subspecific name, *A.triastriatususualis*. In fact, the setal numbers of *A.triastriatususualis* reported by Dong et al. (2020) are closer to those of *A.masatakae* rather than to *A.triastriatus* described by Chen (1946), supporting our conclusion that specimens of Dong et al. (2020) are, indeed, *A.masatakae*.

Shen (2018) criticized the erection of sympatric subspecies since this common, but harmful practice in earthworm taxonomy ignores the biogeographical connotation of the concept. Consistent with this opinion, our results indicate that the distributions of clades A and B overlap substantially and the two clades should not be treated as two distinct subspecies. Additionally, the geographic distributions of these clades and the low genetic variation within each clade suggest that these clades are both peregrine. Clade A has been reported in Korea, China, Japan, Taiwan and India, whereas clade B has been documented in Korea and China. In China, the distributions of the two clades overlap considerably at the national scale, as illustrated in Figure 2 of Dong et al. (2020) and the two clades are even found to co-occur at four of the 35 sampling locations (Dong et al. 2020, their Table 1; see Appendix in Suppl. material 1 for details). Similarly, in Korea, Blakemore (Blakemore 2013a: p. 30 and p. 35) reported sympatry between “*A.masatakae*” (= clade A) and “*A.tralfamadore*” (= clade B). Thus, we conclude that clade A and clade B are intraspecific clades of the same species and *A.tralfamadore* should be considered a junior synonym of *A.masatakae*. Furthermore, the name *Amynthastriastriatususualis* Dong, Jiang, Yuan, Zhao and Qiu, 2020 is nomenclaturally unavailable since the original description of this *A.t.usualis* fails to meet the criteria for electronic publication: To be considered published, a work issued and distributed electronically must be registered in the *Official Register of Zoological Nomenclature* (ZooBank) and contain evidence in the work itself that such registration has occurred (International Commission on Zoological Nomenclature [ICZN] 2012, amended Article 8.5.3.). Moreover, there is no explicit statement on the deposition of the type material (International Commission on Zoological Nomenclature [ICZN] 1999, Article 16.4.2.) and explicit statement that the name “*usualis*” is the new name proposed (International Commission on Zoological Nomenclature [ICZN] 1999, Article 16.1.).

Clade C consists of four sequences of *A.scaberulus* from China, including the holotype of the nominal species. However, *A.scaberulus* described by Sun et al. (2021) is morphologically indistinguishable from *A.tralfamadore* (= *A.masatakae*) described by Blakemore (2012c) (Table 4). Additionally, the COI genetic distance between clades C and A and clades C and B are both in the 9–10% range, suggesting that these three clades can reasonably be considered as members of the same species (Chang et al. 2007, Chang and James 2011, Shen et al. 2016, Shen et al. 2022). Thus, *A.scaberulus* is also a junior synonym of *A.masatakae*. As for clade D, there are no morphological data associated with species identification in the literature; thus, we opted not to speculate.

To sum up, clades A, B and C in our phylogenetic analysis are conspecific and their identity should be *A.masatakae*. This species has frequently been incorrectly identified as *A.robustus*, *A.triastriatus* or *A.gracilis*, as evidenced in many GenBank sequences we analyzed. Molecular data support that *A.masatakae* and *A.robustus* are separate species (Fig. 2) and indicate that *A.tralfamadore* and *A.scaberulus* are junior synonyms of *A.masatakae*. The case of *A.masatakae* is similar to several peregrine earthworm species: they have multiple COI lineages and these lineages often were in sympatry (Rota et al. 2018, Taheri et al. 2018). The diversification of *A.masatakae* led to the publication of *A.tralfamadore* by Blakemore (2012c), *A.triastriatususualis* by Dong et al. (2020) and *A.scaberulus* by Sun et al. (2021). These taxa or lineages are morphologically and distributionally inseparable.

### Distributions of A.masatakae and A.robustus

Our synthesis provided strong evidence that both *A.masatakae* and *A.robustus* are peregrine species mainly found in East Asia. *Amynthasmasatakae* is primarily reported in Japan, Korea and China, with only a few cases in Taiwan and India, whereas *A.robustus* is common in China and Taiwan, rare in Japan (except the Ryukyu Archipelago (Ohfuchi 1956)) and India and absent in Korea. Gates (1939) considered specimens of *A.masatakae* reported in Kobayashi (1937) and Kobayashi (1938) to be *A.robustus* and later wrongly included Korea in the domain of *A.robustus* (Gates 1972). Following false synonymization between *A.masatakae* and *A.robustus* proposed by Ljungström (1972), Easton (1981) incorrectly listed the mainland of Japan as part of the distributional range of *A.robustus*. Nevertheless, in Japan, this species did occur in the Ryukyu Archipelago (Ohfuchi 1956) and was recorded in Godaisan, Kochi, Shikoku after 2000 (Minamiya et al. unpublished data, see GenBank accession nos. AB542526, AB542527 and AB543233), but has not been documented anywhere north of Kyoto.

Both *A.robustus* and *A.masatakae* in these areas have long been confounded with *A.triastriatus* by Chinese authors (e.g., Huang et al. (2007), Dong et al. (2020), Sun et al. (2021)). The original description of *A.triastriatus* is based on a single specimen found in Mt. Omei, Sichuan, central China (Chen 1946). This species appears to be relatively rare as Chen (1933) did not find this species during his extensive survey in the lower Yangtze Valley, central China. In contrast, Dong et al. (2020) not only found “*A.triastriatus*” to be a widely distributed endemic species with a total of 232 individuals collected from 17 provinces in central and southern China, but also drew a comparison of this species to other widespread earthworms, such as *Drawidajaponica*, *Hormogasterelisae*, *Amynthascorticis* and *Amynthasgracilis*. Furthermore, with the inevitable loss of natural habitats, as China rapidly industrialized, it is unlikely for an endemic earthworm species, that was rare 70 years ago, to be common nowadays. Presumably, most, if not all, of the “*A.triastriatus*” records in the last 10–15 years in China are likely *A.masatakae*. Among all the sequences that are currently available in GenBank, none of them can be confirmed to come from a real *A.triastriatus* specimen.

Shen (2018) highlighted that damages caused by misidentification and mis-synonymization can propagate beyond taxonomy, as they provoke not only taxonomic, but also distributional confusion. It is now clear that *A.masatakae* is common in Korea and Japan (Kobayashi 1937, Kobayashi 1938, Ohfuchi 1938, Blakemore 2012c, Blakemore 2013a, Blakemore and Lee 2013), but *A.robustus* is absent or rare with restricted distribution in these areas. In contrast, in Taiwan, *A.robustus* is common (Tsai 1964, Tsai et al. 2009), but *A.masatakae* is rare. These distributional records imply that *A.masatakae* is probably a temperate species, whereas *A.robustus* originated further south. Blakemore (2013a) suggested the provenance of *A.masatakae* from Jeju, South Korea. Phylogeographical inference proposed by Dong et al. (2020) suggested that *A.masatakae* (“*A.triastriatus*” in their article) “originated around Guangxi and Guangdong provinces and generated into two main lineages 2.97 Ma…… at the time of Quaternary glaciation”. Given the genetic structure within *A.masatakae* and the fact that *A.masatakae* is a peregrine species, its modern distribution in China as reported in Dong et al. (2020) most likely resulted from human activities as opposed to natural dispersal and vicariance processes. Thus, the phylogeographical inference proposed by Dong et al. (2020) is unfounded.

### Parthenogenetic polymorphism in A.masatakae

**Figure 3.**
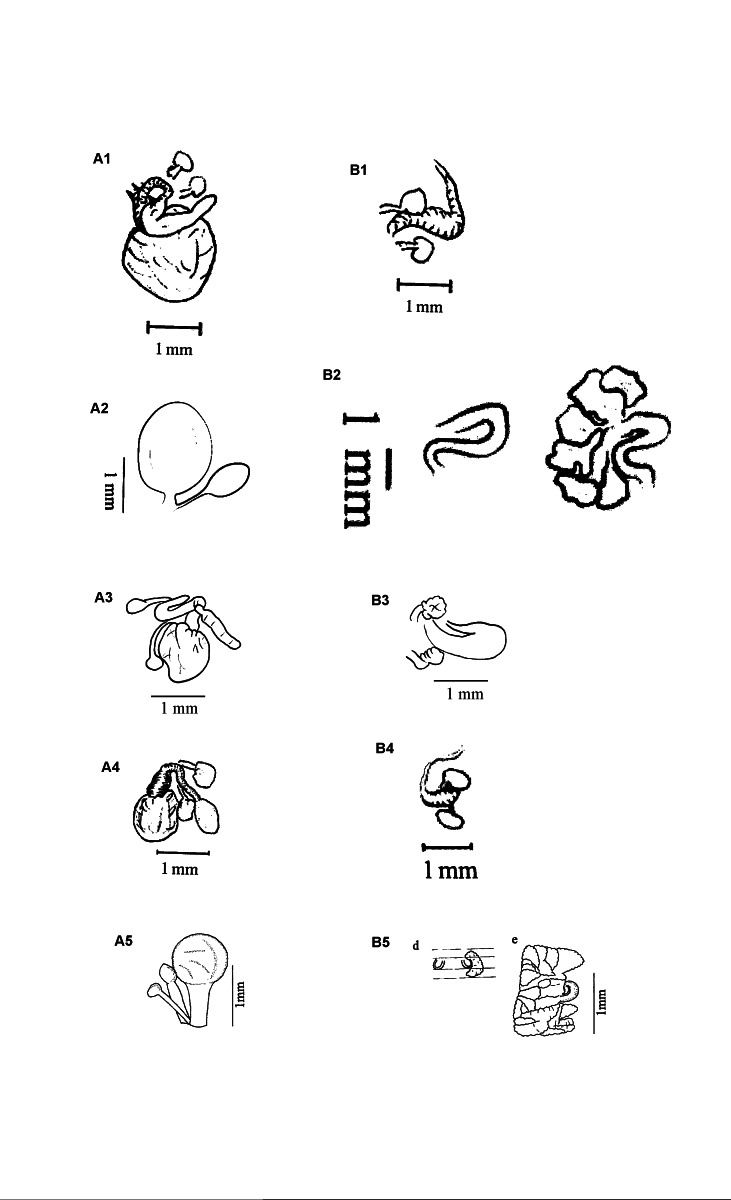
Structures of spermatheca (**A**) and prostate gland (**B**). **A1** Fig. 4A in Blakemore and Lee (2013) modified (voucher number H3); **A2** Figure 8B in Dong et al. (2020); **A3** Specimen from Taiwan (voucher number East 567); **A4** Fig. 7 in Blakemore (2012c) modified (voucher number WO2); **A5** Figure 3f in Sun et al. (2021) (GenBank accession number KF179570); **B1** Fig. 4A in Blakemore and Lee (2013) modified (voucher number H3); **B2** Figure 8C in Dong et al. (2020) modified; **B3** Specimen from Taiwan (voucher number East 567); **B4** Fig. 7 in Blakemore (2012c) modified (voucher number WO2); **B5** Figures 3de in Sun et al. (2021) modified (GenBank accession numbers KF179570 and MF541646).

Together with the newly-collected specimens in Taiwan, our finding that *A.traistriatus* in Dong et al. (2020) is, indeed, *A.masatakae* provides new insight into parthenogenetic polymorphism of the species. Statements in Dong et al. (2020) indicated that clade A has a plump and glossy seminal chamber and small prostate glands and a tendency to parthenogenetic reproduction, whereas clade B has a thin and lusterless seminal chamber and no prostate gland and almost degenerated to parthenogenesis. This difference was used by Dong et al. (2020) to erect a new “subspecies”. However, Blakemore (2012c) showed in his work that the two clades (as “*A.masatakae*” and “*A.tralfamadore*”, respectively) both have no prostate gland and differ only by their shapes of diverticula bulb: paprika-shaped in clade A, but spherical in clade B. Additionally, in clade A, specimens from Taiwan (East 567, this study) and Korea (voucher numbers w28b and H3 in Blakemore (2013a) and Blakemore and Lee (2013), respectively) have cayenne-shaped seminal chambers without iridescence and prostate with duct only (Fig. 3); in clade B, specimens from Korea (Blakemore 2012c, Blakemore 2013a) have a rounded rather than elongated seminal chamber. The various degrees of parthenogenetic degeneration observed in both clades are clear evidence that the morphological differences for erecting a new taxon claimed by Blakemore (2012c) and Dong et al. (2020) are caused by insufficient sampling. Those distinctions do not exist (for more details, see Suppl. material 1).

### Data in GenBank

As the first study in which “DNA barcoding” and “earthworm” appear in the same article, Huang et al. (2007) is the most-cited earthworm DNA barcoding paper and the second most-cited paper of earthworm molecular phylogeny (126 times in Google Scholar as of August 2023). However, unfortunately, it also exemplified the worst of how DNA barcoding can be used or, frankly, abused, in taxonomy. In Huang et al. (2007), an astonishing number of scientific names used are problematic, 10 out of 28 to be exact (Chang et al. 2009). Through carefully re-analyzing COI data, Chang et al. (2009) also found that specimens identified as *A.triastriatus* and *Amynthashawayanus* (Rosa, 1891) (= *A.gracilis*) in Huang et al. (2007) are conspecific and retained the identification of *A.triastriatus* proposed by Huang et al. (2007) owing to the impossibility of determining the exact species identity. It is now clear that the true identity of those specimens is neither *A.gracilis* nor *A.triastriatus*, but *A.masatakae*. In this study, we found that 36 of the 53 sequences of *A.masatakae* are misidentified. This alarming number, which has not included the seven specimens identified as “*A.tralfamadore*” or “*A.scaberulus*”, highlights the importance of an adequate literature review and morphological investigation. Compared with the study by Bridge et al. (2003), which shows that up to 20% of publicly available, taxonomically important DNA sequences for three randomly chosen groups of fungi may be incorrectly named, our study is a strong corroboration of the proliferation of taxonomic misidentification in public DNA databases (Vilgalys 2003).

We urge researchers to be more careful when using sequences in GenBank, especially when relying on DNA barcodes for species identification, and taxonomists who generate sequences to take full responsibility for the sequences they submit to GenBank and to make necessary corrections throughout their academic career. For GenBank users, stop simply relying on the scientific name shown on the sequence page, even when it appears to be identified by a well-known taxonomist. Read the literature of those sequences and other sequences with the same species label and conduct preliminary analyses before deciding which sequences to use. It will always be the responsibility of users to check the identity of specimens and the integrity of their sequence data (Vilgalys 2003). Otherwise, errors associated with incorrect species identification can easily propagate and be magnified through sequence use and re-use by subsequent researchers, which not only causes further confusion, but also exacerbates the chaotic situation.

Taheri et al. (2018) highlighted that sequences of easy access are significant for objectively defining species boundaries, and reproducibility is essential to any scientific result. In our analysis, we were unable to locate 24 out of the 65 COI sequences newly published in Dong et al. (2020), i.e., sequences with GenBank accession numbers starting with “NC” in their Table A1, despite the study being published more than three years ago. We contacted GenBank through email and were notified that “there are currently no such assigned GenBank accessions with the prefix NC”. Although this incidence did not affect our analyses, we feel sad that COI sequence from the purported “holotype” of *A.triastriatususualis* (C‐FJ201111‐04A) with GenBank accession no. NC719760 is among those that are unavailable.

### Species identification of A.masatakae

For species identification of *A.masatakae* in the future, a relatively easy molecular approach is to use three sequences from each of clades A, B and C as reference sequences and conduct a quick phylogenetic analysis (Neighbor-joining, Maximum Likelihood, etc.). Any of the GenBank sequences in clades A, B and C in our phylogenetic tree (Fig. 2) should serve well as reference sequences. As for morphology, researchers should refer to morphological descriptions and illustrations of “*A.masatakae*” by Blakemore (2012c), “*A.triastriatususualis*” by Dong et al. (2020), “*A.tralfamadore*” by Blakemore (2012c) and “*A.scaberulus*” by Sun et al. (2021). We emphasize that researchers should use all of the four sets of descriptions and illustrations as references as opposed to just one or two in order to have a comprehensive picture of the parthenogenetic polymorphism within *A.masatakae*.

## Supplementary Material

79188A91-B8CD-51BA-87BD-9F1A563735CC 10.3897/BDJ.12.e119599.suppl1 Supplementary material 1 Appendix Data type Text file Brief description The issues of sympatric subspecies, distribution of lineages A and B and parthenogenetic polymorphisms reported by Dong et al. (2020). File: oo_965693.doc https://binary.pensoft.net/file/965693 Chang C.-H. et al.

## Figures and Tables

**Table 1.** GenBank accession numbers of COI sequences of specimens used in this study.

Species	Locality	Voucher no.	GenBank accession no.
*Amynthasgracilis* (Kinberg, 1867)	Douliou, Yunlin, western Taiwan	Gra1	OR801254
	Lake Shuangli, Guningtou, Kinmen	Gra2	OR801255
	Central Boulevard, Nangan, Matsu	MTS5	OR801251
	Nanao, Ilan, northeastern Taiwan	East617	OR801252
	Fuhsing, Taoyuan, northern Taiwan	LLS43	OR801253
*Amynthasmasatakae* (Beddard, 1892)	Chilai mountain house, Hualien, eastern Taiwan	East567	OR801241
*Amynthasrobustus* (Perrier, 1872)	Meilan Forest Road, Kaohsiung, southern Taiwan	laut1	OR801247
	Meilan Forest Road, Kaohsiung, southern Taiwan	laut2	OR801248
	Meilan Forest Road, Kaohsiung, southern Taiwan	laut3	OR801249
	Baibaohsi Agricultural Road, Hualien, eastern Taiwan	East348	OR801242
	Lijia Forest Road, Taitung, eastern Taiwan	East382	OR801243
	Fanpaoshan Forest Road, Ilan, northeastern Taiwan	East422	OR801244
	Hubaotan, New Taipei City, northern Taiwan	East686	OR801245
	Wutai, Pingtung, Southern Taiwan	PT001	OR801246
*Perionyxexcavatus* (Perrier, 1872)	Guangfu Forest Road, Hualien, eastern Taiwan	East341	OR801250

**Table 2.** COI sequences published by Blakemore (2012c), Blakemore (2013a) and Blakemore and Lee (2013) and used in this study.

Taxon name in Blakemore’s publication	Source	Locality	Voucher no.
*Amynthastralfamadore* sp. nov.	Blakemore (2012c) and Blakemore and Lee (2013)	Korea	WO2
*Amynthasmasatakae* (Beddard, 1892)	Blakemore (2013a) and Blakemore and Lee (2013)	Japan	WO35
*Amynthasmasatakae* (Beddard, 1892)	Blakemore (2013a) and Blakemore and Lee (2013)	Korea	w28b
*Amynthastralfamadore* Blakemore, 2012	Blakemore (2013a)	Korea	w29
*Amynthastralfamadore* Blakemore, 2012	Blakemore (2013a)	Korea	w30
*Amynthasmasatakae* (Beddard, 1892)	Blakemore and Lee (2013)	Korea	H3

**Table 3.** COI sequences reported in Dong et al. (2020) and used in this study.

Haplotype	Gene code	GenBank accession no.
1	SC18	KF179569
	GZ135	MK225623
3	JX33	MK225617
4	AH60	MK209758
5	GX172	MK225628
6	AH86	MK209755
7	GX210	MK225626
9	GZ151	MK225619
10	AH91	MK225632
11	JX40	MK225616
13	GX189	MK225627
15	AH104	MK225629
16	JX50	MK225615

**Table 4.** A comparison of characters among *A.masatakae* (Beddard, 1892) from Japan, Korea and Taiwan, *A.robustus* (Perrier, 1872) from Taiwan, *A.tralfamadore* Blakemore, 2012 from Korea and *A.triastriatus* (Chen, 1946), *A.triastriatus* “*usualis*” Dong et al. (2020) and *A.scaberulus* Sun and Jiang, 2021 from China.

Species	*A.masatakae*	*A.masatakae*	*A.masatakae*	*A.masatakae*	*A.robustus* as *Pheretimalauta*	*A.tralfamadore*	*A.scaberulus*	*A.triastriatus* “*usualis*”	*A.triastriatus*
Source	Beddard (1892)	Ohfuchi (1938)	Kobayashi (1938)	Chuang and Chen (2002)	Tsai (1964)	Blakemore (2012c)	Sun et al. (2021)	Dong et al. (2020)	Chen (1946)
Locality	Japan	Japan	Korea	Taiwan	Taiwan	Korea	China	China	China
Length (mm)	127	135–189	102–130	105–138	125–203	125	129–166	120–150	110
Segments	90	110–125	114–130	96–138	88–129	125	129–131	108–111	88
Diameter (mm)	6	4.5–7	5.5–7	4–7.5	6.5–7.5	–	4.5–6	4.9–6.8	7
First dorsal pore	–	12/13	11/12	11/12	11/12	10/11	10/11 or 11/12	11/12	10/11
Setal number									
III	–	–	20–30	–	–	–	21–38	20–26	34
VI	–	34–36	39–45 (VII)	34–41 (VII)	–	–	36–42 (V)	24–30 (V)	–
VIII	–	40–43	40–50	–	47–55	–	46–52	30–34	36 (IX)
XII	–	–	47–57	–	–	46–50	–	–	–
XX	–	44–51	54–64	41–49	–	ca. 70	56–62	40–48	–
XXV	–	–	–	–	55 (XXVI)	ca. 70	52–74	55–60	38
Between male pores	–	8–9	13–15	13–15	18–21	ca. 15	14–20	12–16	12
Genital papillae									
Preclitellar	VIII and IX (= 2 medial to each spermathecal pore)	1 medial to each spermathecal pore	2 medial to each spermathecal pore	2 medial to each spermathecal pore	1 medial to each spermathecal pore and paired presetal on ventral VIII–IX	2 medial to each spermathecal pore	1–2 medial to each spermathecal pore	14–18 from postsetal VII to presetal IX	2 medial to each spermathecal pore
Postclitellar	–	2 medial to each male pore	2 medial to each male pore	2 medial to each male pore	1–2 medial to each male pore and 1–2 presetal on ventral XVIII	2 medial to each male pore	1–2 medial to each male pore	2 medial to each male pore and 2 presetal on ventral XVIII	2 medial to each male pore
Spermathecal pores	2 pairs (7/8/9)	2 pairs (7/8/9)	2 pairs (7/8/9)	2 pairs (7/8/9)	2 pairs (7/8/9)	2 pairs (7/8/9)	2 pairs (7/8/9)	2 pairs (7/8/9)	2 pairs (7/8/9)
Spermathecae	small, diverticulum longer than ampulla	ampulla 1.5 mm long, diverticulum much longer than ampulla, no seminal chamber	ampulla small, diverticulum longer than ampulla with distended ental end forming a large seminal chamber	ampulla oval-shaped, diverticulum with a slender stalk and a long seminal chamber	ampulla large, diverticulum long with a slender duct and a rod-like seminal chamber	ampulla round on short duct with rounded clavate diverticulum	ampulla ball-shaped, stout duct as long as ampulla; diverticulum shorter than the main axis, distal ⅓ dilated into a peach-shaped seminal chamber	ampulla oval‐shaped, diverticulum short with terminal 1/2 dilated into an oval‐shaped glossy seminal chamber	ampulla large, heart-shaped, diverticulum long, with ental half enlarged as seminal chamber
Hearts	–	–	4 pairs in X–XIII	–	–	4 pairs in X–XIII	4 pairs in X–XIII	4 pairs in X–XIII	4 pairs in X–XIII
Intestinal caeca	XXVI	XXVIII, serriformed ventral margin	XXVI, simple without indentations	XXVII, simple with saw-shaped border	XXVI, simple with serrated ventral border	XXVII, simple in having a paler rugose and capillaried interior face	XXVII, simple, or with tiny incisions on ventral margins	XXVII, simple with short pointed saccules on ventral margin	simple, smooth
Seminal vesicles	small in XI–XII	weak and thin in XI–XII	small in XI–XII	large in XI–XII	XI–XII	larger in XI and smaller in XII	underdeveloped in XI–XII	XI–XII, second pair more developed	small in XI–XII
Prostate glands	absent	absent	absent or rudimentary	absent or rudimentary	XVI–XIX	aborted	degenerated or developed	small, rudimentary or absent	absent
Prostatic ducts	short and bent muscular	large, muscular, bent	small, bow-shaped	slender, bent	long, slender, bent	short, muscular	bent	S-shaped	U-shaped, stout
Accessory glands	pear-shaped	large, long-stalked	large, long-stalked	–	stalked	small, stalked	stalked	–	stalked

**Table 5.** Uncorrected *p*-distances (lower-left) and Kimura’s two-parameter distances (upper-right) among clades A–E, based on the COI gene.

	Clade A	Clade B	Clade C	Clade D	Clade E
Clade A	–	0.066	0.103	0.105	0.167
Clade B	0.062	–	0.100	0.110	0.169
Clade C	0.094	0.092	–	0.125	0.180
Clade D	0.097	0.100	0.113	–	0.161
Clade E	0.148	0.150	0.159	0.144	–
