# Supplementary material for: A review of the earthworm Amynthasmasatakae (Beddard, 1892) (Clitellata, Megascolecidae), with designation of two new synonyms
Source: Biodivers Data J. 2024 May 9;12:e119599. doi: 10.3897/BDJ.12.e119599 (PMC11099468; doi:10.3897/BDJ.12.e119599)
Supplement: Supplementary material 1 — Appendix [file bdj-12-e119599-s001.doc]

**Appendix**

**The issues of sympatric subspecies, distribution of lineages A and B, and parthenogenetic polymorphisms reported by Dong et al. (2020)**

Dong et al. (2020: p. 18, second paragraph) claimed that the two lineages, A and B, distribute differently with the former mainly at high altitudes in southwest China while the latter at low altitudes in southeast China. It was also mentioned that a high degree of isolation and a great genetic differentiation exist between the two lineages (Dong et al. 2020: p. 17). Eighteen locations of lineage A and seventeen locations of lineage B were presented in Figure 1 and Table 1 by Dong et al. (2020). Nevertheless, among the 35 localities where specimens were collected, it was found that the two lineages were collected at the same spot in four localities (A4B5, A5B6, A8B7, and A18B14). Even Dong et al. (2020: p. 18) made the following statement: “For example, the locations of A4 and B5, A8 and B7, A18 and B14 overlapped”. Therefore, the actual number of collection sites is 31, not “35 different locations” as mentioned in the materials and methods section by Dong et al. (2020: p. 2). Additionally, localities A3 and B4 were found to be within 1 km apart (actual distance about 937 m), and distance between the following localities is shorter than 3 km according to coordinates given in Table 1 of Dong et al. (2020): A11 and B8 (actual distance about 1125 m), A15 and B9 (actual distance about 2562 m), and A17 and B12 (actual distance about 1437 m).

Lineages A and B were further divided into four (clades 1–4) and two (clades 5 and 6) clades, respectively, by Dong et al. (2020). Figure 2 of Dong et al. (2020) shows that distributional areas of clades 1 and 4 overlap with those of clade 6, and those of clades 2 and 3 with those of clade 5. Meanwhile, on the basis of Table 1 by Dong et al. (2020), elevations for lineage A are 93–1625 m and those for lineage B are 120–1558 m. Obviously, distributional ranges as well as elevations of lineages A and B greatly overlap. Accordingly, data presented by Dong et al. (2020) are inconsistent with statements made by the same authors.

Dong et al. (2020: p. 17) stated that the erection of a new subspecies was based on certain morphological differences between the two lineages. According to Dong et al. (2020: p. 18, second paragraph), lineage A with a thin and lustreless seminal chamber and no prostate gland observed was almost degenerated to parthenogenesis, while lineage B with a plump and glossy seminal chamber and small prostate glands had a tendency to parthenogenetic reproduction. Our phylogenetic tree demonstrates that our clade A includes voucher numbers East 567 from Taiwan and w28b and H3 from Korea (Blakemore 2013, Blakemore and Lee 2013), as well as COI sequences of lineage B from China reported by Dong et al. (2020) (GenBank accession numbers MK225615 (gene code: JX50, haplotype 16) and MK225629 (gene code: AH104, haplotype 15)). Specimens from both Taiwan and Korea (Blakemore 2013, Fig. 9A, Blakemore and Lee 2013, Fig. 4A) have cayenne-shaped seminal chamber without iridescence and prostate with duct only (see Fig. 3 of the main text). On the other hand, our clade B includes voucher numbers WO2, w29, and w30 from Korea reported by Blakemore (2012, 2013), as well as COI sequences of lineage A from China reported by Dong et al. (2020) (GenBank accession numbers MK225627 (gene code: GX189, haplotype 13), MK225623 (gene code: GZ135, haplotype 1), KF179569 (gene code: SC18, haplotype 1), MK225617 (gene code: JX33, haplotype 3), MK209755 (gene code: AH86, haplotype 6), MK209758 (gene code: AH60, haplotype 4), MK225628 (gene code: GX172,, haplotype 5), MK225626 (gene code: GX210, haplotype 7), MK225616 (gene code: JX40, haplotype 11), MK225632 (gene code: AH91, haplotype 10), and MK225619 (gene code: GZ151, haplotype 9)). Specimens from Korea have rounded rather than elongated seminal chamber (Blakemore 2012, Fig. 7, 2013, Fig. 9B). Evidently, different degrees of parthenogenetic degeneration can be observed in either of the clades and this is contradictory to Dong et al.’s (2020) dichotomous statement.

The morphological distinctiveness for the erection of a new taxon claimed by Blakemore (2012) and Dong et al. (2020) is undoubtedly due to insufficient sampling. Albeit Dong et al. (2020: p. 18, second paragraph) compared morphological and distributional differences between lineages A and B, these differences, as elaborated above and in the main text, do not exist. Also, it is irrational that these comparisons lead to the following conclusion by Dong et al. (2020: p. 18, second paragraph): “These findings supported those of Simon et al. (2003), that unisexual lineages with high genetic diversity can live in wider geographical ranges than their sexual ancestor.” The identity as well as the existence of the sexual ancestor is unknown, let alone its geographical range. Dong et al. (2020: p. 18, third paragraph) further claimed that the later divergence of lineage A than lineage B indicated the derivation of the former from the latter. Apparently, Dong et al. (2020) mistook lineage B for the sexual ancestor.

**References**

Blakemore RJ (2012) New earthworm species from NIBR’s Jeju-do biosphere compared to historical and new Japanese types (Oligochaeta: Megadrilacea: Megascolecidae). Journal of Species Research 1 (2): 133-150. https://doi.org/10.12651/JSR.2012.1.2.133

Blakemore RJ (2013) Jeju-do earthworms (Oligochaeta: Megadrilacea)-Quelpart Island revisited. Journal of Species Research 2 (1): 15-54. https://doi.org/10.12651/JSR.2013.2.1.015

Blakemore RJ, Lee S (2013) Survey of Busan Oligochaeta earthworms supported by DNA barcodes. Journal of Species Research 2 (2): 127-144. https://doi.org/10.12651/JSR.2013.2.2.127

Dong Y, Jiang J, Yuan Z, Zhao Q, Qiu J (2020) Population genetic structure reveals two lineages of *Amynthas triastriatus* (Oligochaeta: Megascolecidae) in China, with notes on a new subspecies of *Amynthas triastriatus*. International Journal of Environmental Research and Public Health 17: 1538. https://doi.org/10.3390/ijerph17051538
